# Readmissions performance and penalty experience of safety-net hospitals under Medicare’s Hospital Readmissions Reduction Program

**DOI:** 10.1186/s12913-022-07741-9

**Published:** 2022-03-15

**Authors:** Souvik Banerjee, Michael K. Paasche-Orlow, Danny McCormick, Meng-Yun Lin, Amresh D. Hanchate

**Affiliations:** 1 Department of Humanities and Social Sciences, Indian Institute of Technology Bombay, Mumbai, Maharashtra India; 2 Section of General Internal Medicine, Boston University School of Medicine, Boston, MA USA; 3 Boston Medical Center, Boston, MA USA; 4 Harvard Medical School, Boston, USA; 5 Division of Social and Community Medicine, Department of Medicine, Cambridge Health Alliance, Cambridge, MA USA; 6 Department of Social Sciences and Health Policy, Wake Forest School of Medicine, Medical Center Boulevard, Winston-Salem, NC 27157-1063 USA; 7 Division of Public Health Sciences, Department of Social Sciences and Health Policy, Wake Forest School of Medicine, Medical Center Boulevard, Winston-Salem, NC 27157-1063 USA

**Keywords:** Hospital performance, Readmissions, Penalty, Safety-net hospitals

## Abstract

**Background:**

The Hospital Readmissions Reduction Program (HRRP), established by the Centers for Medicare and Medicaid Services (CMS) in March 2010, introduced payment-reduction penalties on acute care hospitals with higher-than-expected readmission rates for acute myocardial infarction (AMI), heart failure, and pneumonia. There is concern that hospitals serving large numbers of low-income and uninsured patients (safety-net hospitals) are at greater risk of higher readmissions and penalties, often due to factors that are likely outside the hospital’s control. Using publicly reported data, we compared the readmissions performance and penalty experience among safety-net and non-safety-net hospitals.

**Methods:**

We used nationwide hospital level data for 2009-2016 from the Centers for Medicare and Medicaid Services (CMS) Hospital Compare program, CMS Final Impact Rule, and the American Hospital Association Annual Survey. We identified as safety-net hospitals the top quartile of hospitals in terms of the proportion of patients receiving income-based public benefits. Using a quasi-experimental difference-in-differences approach based on the comparison of pre- vs. post-HRRP changes in (risk-adjusted) 30-day readmission rate in safety-net and non-safety-net hospitals, we estimated the change in readmissions rate associated with HRRP. We also compared the penalty frequency among safety-net and non-safety-net hospitals.

**Results:**

Our study cohort included 1915 hospitals, of which 479 were safety-net hospitals. At baseline (2009), safety-net hospitals had a slightly higher readmission rate compared to non-safety net hospitals for all three conditions: AMI, 20.3% vs. 19.8% (*p* value< 0.001); heart failure, 25.2% vs. 24.2% (*p*-value< 0.001); pneumonia, 18.7% vs. 18.1% (*p*-value< 0.001). Beginning in 2012, readmission rates declined similarly in both hospital groups for all three cohorts. Based on difference-in-differences analysis, HRRP was associated with similar change in the readmissions rate in safety-net and non-safety-net hospitals for AMI and heart failure. For the pneumonia cohort, we found a larger reduction (0.23%; *p* < 0.001) in safety-net hospitals. The frequency of readmissions penalty was higher among safety-net hospitals. The proportion of hospitals penalized during all four post-HRRP years was 72% among safety-net and 59% among non-safety-net hospitals.

**Conclusions:**

Our results lend support to the concerns of disproportionately higher risk of performance-based penalty on safety-net hospitals.

**Supplementary Information:**

The online version contains supplementary material available at 10.1186/s12913-022-07741-9.

The Hospital Readmissions Reduction Program (HRRP), enacted as part of the 2010 Affordable Care Act, aimed to reduce preventable hospital readmissions as a way to lower inpatient costs without compromising quality of care [1, 2]. Under HRRP, the Centers for Medicare and Medicaid Services (CMS), was mandated to review Medicare fee-for-service (FFS) hospitals paid under the Medicare Inpatient Prospective Payment System (IPPS) and impose financial penalties on the hospitals that exhibited higher-than-expected 30-day readmission rate for specified clinical conditions [3]. Financial penalties, which took the form of a percentage reduction in Medicare payments for a hospital’s inpatient care services, were levied on IPPS hospitals for excess readmissions starting October 1, 2012 (fiscal year (FY) 2013), with up to 1% of qualifying hospitals’ total Medicare reimbursements being withheld in the first year; the maximum penalty increased to 3% in FY 2015 and has been capped at this level [1].

Many observers have expressed concern about the program since it increases the risk of penalty for hospitals that serve higher proportions of uninsured, low income, and medically vulnerable patients – that is, safety-net hospitals – and therefore could adversely affect the financial viability of the hospitals and the services to the poor and vulnerable patients [4, 5]. Further, risk of penalty for such hospitals may be unduly higher since many factors that are associated with higher risk of readmission – inadequate social supports, low income, low levels of education, residential instability, risk health behaviors – are not included in the risk adjustment model for determining hospital performance [6–12].

There is a growing literature on assessing the impact of HRRP on the penalty experience and on readmissions. Early studies suggest that safety-net hospitals were penalized at a higher rate and incurred higher penalty under HRRP compared to non-safety-net hospitals [3, 13–15]. These studies documenting the penalty experience were limited by single state [14], single year [13] or single measure [15]. On the impact on readmissions performance, studies have varied on methodology and data used, thereby limiting comparability. One study assessed the differential impact on the readmissions rate in safety-net vs. non-safety-net hospitals [16]; however, as the analysis did not include pre-HRRP data, it did not adjust for baseline differences across hospitals. Another study used a hospital-wide readmission measure to compare safety-net vs. non-safety hospitals under HRRP [17]; however, this measure uses different patient cohorts and risk measures than that used by CMS in determining hospital performance [18]. To provide a more comprehensive and authentic assessment of the readmissions and penalty experience, what is needed is a nationally representative sample, with performance data from pre- and post-program periods for the same patient cohorts and using the same risk-adjustment method used by HRRP [1, 19].

In this study, we improved upon prior work by using pre-HRRP data in addition to post-HRRP data and examined whether changes in readmission rates associated with HRRP were different for safety-net vs. non-safety-net hospitals for the three targeted conditions – AMI, heart failure, and pneumonia. Specifically, we compared changes in readmission rates in the post- vs. pre-HRRP period for safety-net hospitals with corresponding changes for non-safety-net hospitals using publicly reported hospital-level data from 2009 to 2016. In addition, we examined multiple penalty measures – share of hospitals penalized, average penalty, and distribution of repeated penalties – aimed at capturing the penalty experience of safety-net vs. non-safety-net hospitals from 2013 to 2016. Although changes are currently under way, whereby CMS will evaluate hospital performance relative to other hospitals with a similar share of dually eligible Medicare and Medicaid patients starting fiscal year (FY) 2019, understanding the impact of the initial years of HRRP on safety-net hospitals informs future interventions target hospital quality [20, 21].

## Methods

### Data and analysis

We obtained data on hospital readmissions performance (2009-2016) from CMS’ Hospital Compare Program [22] and data on safety-net hospital status (2009) and penalty (2013-2016) from CMS’ Final Impact Rule [23]. In addition, we used the American Hospital Association Annual Survey (2009) to obtain data on hospital characteristics [24]. Our sample universe included all IPPS hospitals over the period 2009-2016, with 2756 such hospitals in 2009 to 2607 in 2016. Non-IPPS hospitals were excluded from the analysis; these included all Maryland hospitals, critical access hospitals, pediatric hospitals, long-term care facilities, rehabilitation hospitals, psychiatric hospitals, and Veterans Affairs hospitals [25]. We also excluded hospitals that did not report 30-day-risk-adjusted readmissions or were not reimbursed through the IPPS during all the study years.

### Readmission outcomes

Our analytic data were comprised of longitudinal (annual) observations for the included IPPS hospitals from 2009 to 2016. Our main outcomes were 30-day risk-adjusted readmission rates for the three conditions targeted by HRRP: AMI, heart failure, and pneumonia. For each hospital, the Hospital Compare Program reports 30-day risk-adjusted readmission rates for each cohort in each year, based on eligible admissions in the preceding 3 years and is adjusted for differences between hospitals in patient characteristics, including age, sex, comorbid health conditions, as well as other unobserved, systematic hospital effects [19, 26]. Therefore, the 30-day risk-adjusted readmission rate for 2009 corresponds to data collection period July, 2005-June, 2008 and similarly for the other years (Additional file 1: Table 1). We restricted the sample for the pneumonia cohort to 2009-2015, since CMS’ definition of pneumonia for this measure was modified in 2016 and resulted in a large increase in the number of eligible admissions [19].

### Safety-net hospitals

We followed prior studies and used the disproportion share hospital (DSH) index to identify safety-net hospitals. The DSH index is defined as the sum of the proportion of elderly patients who receive Supplemental Security Income (SSI) and the proportion of non-elderly patients who receive Medicaid benefits [27, 28]. We defined the top quartile of hospitals in terms of the DSH index as safety-net hospitals. There is no single universally accepted method for identifying safety-net hospitals, and some of the alternatives that have been used include: Medicaid caseload, uncompensated care burden, and facility characteristics, with each having its own merits [14]. We prefer the DSH index to define safety-net hospitals since it is able to identify poor patients regardless of their age [14, 28]. Nonetheless, in sensitivity analysis we used the share of aggregate inpatient days attributable to Medicaid patients out of aggregate inpatients days for all patients as an alternative measure.

### Hospital characteristics

We included several hospital characteristics in our analyses that were available from the American Hospital Association Annual Survey. These were bed size (less than 100, 100-199, and 200 or more); teaching hospital status based on membership in the Council of Teaching Hospitals; ownership (not-for-profit, government non-federal, and for-profit), region (Northeast, Midwest, South, and West), share of Medicare inpatient days out of total inpatient days, and share of Medicaid inpatient days out of total inpatient days.

### Analysis

The hospital characteristics of safety-net vs. non-safety-net hospitals were compared in the baseline year 2009. We conducted t-tests for differences between the two groups for continuous variables and chi-square tests for categorical variables. We plotted trends in hospital readmission rates for each condition for safety-net vs. non-safety-net hospitals over the study period. Linear time series models were estimated to capture average annual change in readmission rates for the same conditions from 2009 to 2016 [29].

The association between the main outcome (risk-adjusted hospital 30-day readmission rate for each condition) and safety-net hospital status was estimated using a difference-in-differences approach, whereby pre- vs. post-period changes in the outcome were compared between safety-net hospitals and non-safety-net hospitals [30, 31]. Since HRRP was announced in March 2010, we considered hospital readmissions performance in and after 2010 as potentially influenced by HRRP (i.e., post-period), even though the first year of implementation was 2013. As noted, the Hospital Compare readmission rates reported were based on admission during the three preceding years. Accordingly, we categorized the readmission rates for 2009 and 2010 (for admissions during July 2005 to June 2009) as representing the pre-HRRP period, and the rates for 2014 to 2016 (for admissions during July 2010 to July 2015) as representing the post-HRRP period. The intervening years 2011-2013 (the “washout” period) were excluded from the difference-in-differences analysis since the readmission rates were based on both pre- and post-2010 time period.

For our core analysis to estimate the change in the main outcome (risk-adjusted 30-day readmission rate) associated with HRRP we used a linear hospital-level random effects regression model with a difference-in-differences specification and heteroscedasticity-robust standard errors [32, 33]. For the difference-in-differences specification, we included an indicator for the safety-net hospitals, a post-period time indicator, an interaction term between safety-net hospitals and post-period – the difference-in-differences estimator – and adjusted for hospital characteristics in the baseline year. The difference-in-differences estimate gives the excess pre to post change in readmission rate for safety-net hospitals compared to that for non-safety-net hospitals [30, 34, 35]. The difference-in-differences approach assumes similarity in pre-period trends for each outcome between safety-net and non-safety net hospitals (“parallel trends assumption”) [31]. We tested for parallel trends by estimating a placebo version of the proposed difference-in-difference models using only pre-period data (2009-2010) (Additional file 1: Table 2). Specifically, outcome in 2009 was compared with that in 2010 (“post 2010”). Absence of a significant coefficient of the interaction term (safety-net x post) is indicative of similar trends in safety-net and non-safety-net hospitals. We also examined sensitivity of the estimates to an alternative longitudinal data structure model that controls for time-invariant unobserved differences across hospitals (i.e., “hospital fixed effects”) [33, 36]. All models included year fixed effects to adjust for secular trends in readmission rates.

Using data on HRRP penalties from 2013 to 2016, we compared several indicators of the penalty experience of safety-net vs. non-safety-net hospitals: (i) share of hospitals penalized, (ii) average annual penalty, and (iii) distribution of repeated penalties. The comparisons were made using t-test.

Statistical analyses were conducted using Stata version 14.1 [37]. The Institutional Review Board of the Boston University School of Medicine considered this study exempt from human subjects review as no person-level data was involved.

## Results

Our final analytic sample included 1915 hospitals in each year from 2009 to 2016. The characteristics of the safety-net and non-safety-net hospitals are shown in Table 1. The mean DSH index value was 0.54 for safety-net hospitals and 0.20 for non-safety-net hospitals. Safety-net hospitals were more likely to be teaching hospitals (25%) than non-safety-net hospitals (8%). Safety-net hospitals had a lower share of Medicare and higher share of Medicaid inpatient days relative to non-safety-net hospitals (Medicare share: 0.41 vs 0.52; Medicaid share: 0.29 vs. 0.15). The majority of the safety-net hospitals were concentrated in the South and West (72.02% overall) and non-safety-net hospitals in the Midwest and South (63.37% overall).

**Table 1:** Comparison of hospital characteristics of safety-net hospitals vs. non-safety-net hospitals, 2009

	Safety-net hospitals		Non-safety-net hospitals		*P* value^§^ (safety-net vs. non-safety-net hospitals)
	(*n* = 479)		(*n* = 1436)		
	n	%	n	%	
Disproportionate Share Hospital index: Mean (Standard Deviation)	0.54 (0.082)		0.20 (0.173)		< 0.001
Teaching hospital^a^	120	25.05	121	8.43	< 0.001
Ownership					< 0.001
Non-profit	250	52.19	1042	72.56	
Govt. non-fed	121	25.26	141	9.82	
For-profit	108	22.55	253	17.62	
Medicare share inpatient days Mean (Standard Deviation)	0.41 (0.006)		0.52 (0.003)		< 0.001
Medicaid share inpatient days Mean (Standard Deviation)	0.29 (0.006)		0.15 (0.002)		< 0.001
Bed size					0.001
< 99	63	13.15	270	18.80	
100-199	119	24.84	415	28.90	
> =200	297	62.00	751	52.30	
Region					< 0.001
Northeast	72	15.03	301	20.96	
Midwest	62	12.94	395	27.51	
South	200	41.75	515	35.86	
West	145	30.27	225	15.67	

1) Hospitals appearing in either the AMI, heart failure, or pneumonia cohort are included for the findings in this table

2) Safety-net hospitals: hospitals that fall in the top quartile of the Disproportionate Share Hospital (DSH) index; non-safety-net hospitals: hospitals in the bottom three quartiles of the DSH index

3) §t-test for continuous variables and chi-square test for categorical variables

4) ^a^Member of Council of Teaching Hospital of the Association of American Medical Colleges

**Fig. 1:**
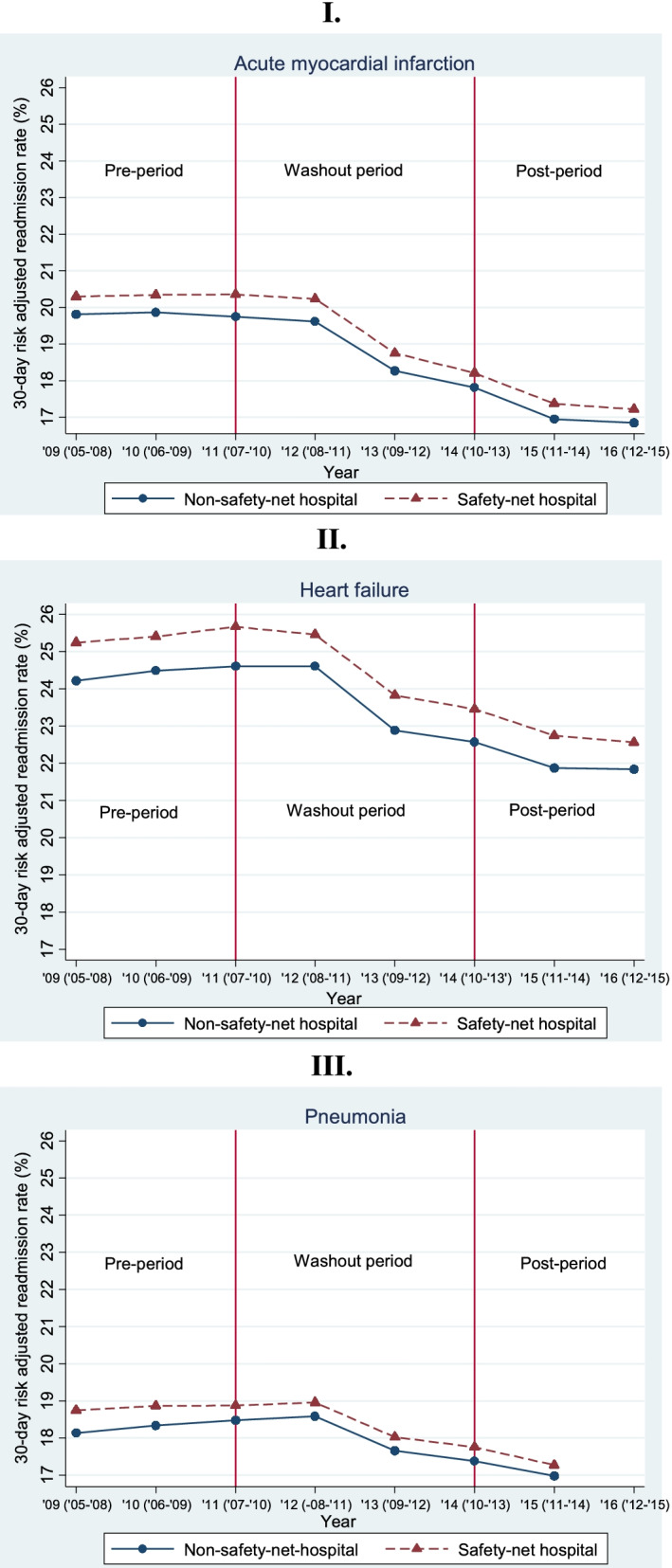
Average hospital 30-day risk adjusted readmission rate (%) by safety-net status and admission condition, 2009-2016. Notes: Safety-net hospitals: hospitals that fall in the top quartile of the Disproportionate Share Hospital (DSH) index; non-safety-net hospitals: hospitals in the bottom three quartiles of the DSH index. Pre-period: FY 2009-2010; Washout period: FY 2011-2013; Post-period: FY 2014-2016. Data reporting year and data collection period (in parenthesis) reported from Hospital Compare website

Figure 1 indicates the longitudinal 30-day risk adjusted readmission rates for safety-net vs. non-safety hospitals by admission condition. In 2009, the baseline year, safety-net hospitals had a slightly higher readmission rate compared to non-safety net hospitals for all three conditions: AMI, 20.3% vs. 19.8% (*p* value< 0.001); heart failure, 25.2% vs. 24.2% (*p*-value< 0.001); pneumonia, 18.7% vs. 18.1% (*p*-value< 0.001). Beginning in 2012, readmission rates declined for both hospital groups for all three conditions. The average (unadjusted) annual change in readmission rates for safety-net vs. non-safety-net hospitals over the study period was − 0.51% vs. -0.53% [*p*-value = 0.80] for AMI, − 0.45% vs. -0.48% [*p*-value = 0.74] for heart failure, and − 0.22% vs. -0.27% [*p*-value = 0.59] for pneumonia.

To test the validity of the proposed difference-in-differences approach to obtain adjusted rates of post-program change in readmission rates, we compared pre-period readmission trends (using 2009-2010 data) between safety-net vs. non-safety-net hospitals (“parallel trends test”) and found that for all three admission cohorts pre-period trends were similar among both the hospital groups (Additional file 1: Table 2). Applying this approach to the analytic data spanning the pre and post HRRP periods we found that pre-to-post change in readmission rates were similar among safety-net and non-safety-net hospitals for AMI and heart failure (Table 2; Additional file 1: Table 3). For pneumonia admissions, we found a larger post-period reduction (of 0.23%) in safety-net hospitals relative to non-safety-net hospitals (Additional file 1: Table 3). In sensitivity analysis using an alternative approach to identify safety-net hospitals, based on Medicaid share of total hospital inpatient days of care, we found largely similar results of pre-to-post adjusted changes in readmission rates (Additional file 1: Table 4). Estimation using a hospital-level fixed effects specification also yielded similar results (Additional file 1: Table 5).

**Table 2:** Difference in 30-day risk adjusted readmission rates between safety-net and non-safety-net hospitals, 2009-2010 and 2014-2016

	30-day risk adjusted readmission rate (%)	
	Safety-net hospitals	Non-safety-net hospitals
**A. Acute myocardial infarction**		
Pre-period	20.3	19.8
Post-period	17.6	17.2
Pre to Post Difference	−2.7***	− 2.6***
Difference-in-differences	− 0.08	
**B. Heart failure**		
Pre-period	25.3	24.3
Post-period	22.9	22.1
Pre to Post Difference	−2.4***	−2.3***
Difference-in-differences	−0.14	
**C. Pneumonia**		
Pre-period	18.8	18.2
Post-period	17.5	17.2
Pre to Post Difference	−1.3***	−1.1***
Difference-in-differences	−0.23***	

1) Safety-net hospitals: hospitals that fall in the top quartile of the Disproportionate Share Hospital (DSH) index; non-safety-net hospitals: hospitals in the bottom three quartiles of the DSH index

2) Pre-period denotes the year 2009-2010; post-period denotes the period 2014-2016

3) The sample sizes for the AMI, heart failure, and pneumonia cohorts are 7225; 9370 and 7580 hospital-years, respectively

4) Observed average readmission rates reported for pre-period and post-period. Difference in average readmission rate between post vs. pre period reported for safety-net and non-safety-net hospitals based on linear random effects model regressing mortality rate on post-period indicator; heteroscedasticity-robust standard errors clustered at the hospital level

5) Difference-in-differences estimates from random effects model reported; heteroscedasticity-robust standard errors clustered at the hospital level; covariates in the model include teaching hospital status, ownership, bed size, year and region. Full model estimates are reported in Additional file 1: Table 3. Note that Medicaid and Medicare share of aggregate inpatient days (Table 1) are not included as covariates since they are likely to be associated with the categorization of hospitals into safety-net and non-safety-net hospitals

6) *** *p* < 0.01

**Fig. 2:**
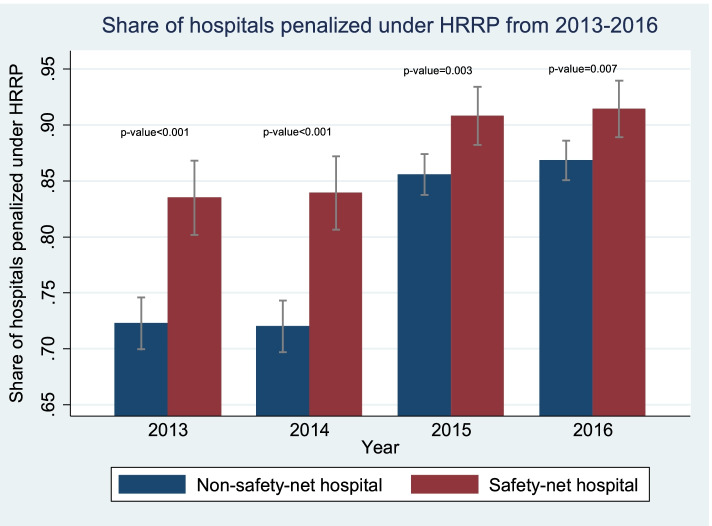
Share of hospitals penalized under HRRP by safety-net status, 2013-2016. Notes: 1) Safety-net hospitals: hospitals that fall in the top quartile of the Disproportionate Share Hospital (DSH) index; non-safety-net hospitals: hospitals in the bottom three quartiles of the DSH index. 95% confidence intervals indicated with vertical lines. 2) *p*-value for difference in mean share of hospitals penalized under HRRP between safety-net and non-safety-net hospitals reported for each year 2013-2016

The proportion of hospitals penalized under HRRP was significantly higher among safety-net hospitals than non-safety-net hospitals in each year of the program (Fig. 2); in 2016, 90% of safety-net hospitals were penalized, compared to 85% of non-safety-net hospitals. The proportion of safety-net hospitals (72%) that were penalized all 4 years was higher compared to that for non-safety-net hospitals (59%) (Additional file 1: Fig. 3). In the first 2 years, the average penalty rate was also higher for safety-net hospitals (Table 3).

**Table 3:** Average penalty under HRRP by safety-net status, 2013-2016

	Safety-net hospitals (*N* = 479)	Non-safety-net hospitals (*N* = 1436)	*P*-value
	%	%	
2013	0.37	0.28	< 0.001
2014	0.34	0.25	< 0.001
2015	0.49	0.52	0.296
2016	0.48	0.52	0.207

1) Safety-net hospitals: hospitals that fall in the top quartile of the Disproportionate Share Hospital (DSH) index; non-safety-net hospitals: hospitals in the bottom three quartiles of the DSH index

2) *p*-value for difference in average penalty under HRRP between safety-net and non-safety-net hospitals reported for each year, 2013-2016. The reported average penalty is on the proportion of total annual Medicare payments received by the hospital

## Discussion

Our study highlights two contrasting findings on the experience of safety-net hospitals following HRRP. Readmission rates for all three admission conditions declined, and the extent of reduction in safety-net hospitals was no smaller than that in non-safety hospitals. Specifically, we found similarity in reduction for AMI and heart failure cohorts; although safety-net hospitals experienced a larger reduction for the pneumonia cohort compared to non-safety-net hospitals the margin of difference was small (0.23% larger reduction over a 18.8% baseline rate). However, the proportion of safety-net hospitals penalized under HRRP was higher than that among non-safety-net hospitals during each of the 4 years examined (2013-2016).

This study extends prior work on HRRP impact on safety-net hospitals by addressing methodological differences in previous studies that limit the comparability and interpretability of findings: we contrasted within hospital changes in post-program with pre-program readmission rates; we used readmission rates obtained from the Hospital Compare program instead of those derived from alternative admission cohorts or risk adjustment methods; we examined a longer post-program period. Our finding that readmission rates in safety-net hospitals decreased at least as much as in non-safety-net hospitals for all three admission conditions is largely in concordance with the findings of the previous studies. The study by Carey and Lin [16] was based on comparison of only the post-program experience of the hospitals. Specifically, comparing change in readmission rates between 2013 and 2016 in safety-net hospitals with that in (all or a matched subgroup of) non-safety-net hospitals, that study found similar changes in both settings or modestly higher reductions in safety-net hospitals across the different conditions [16]. Salerno et al. [17] compared readmission rates for (nearly) all hospitalized patients, and found that between 2008 and 2015, readmission rates in safety-net hospitals decreased more than in the non-safety-net hospitals, although the magnitude of the difference was small. Our study uses hospital readmission performance during 2010 to 2015 to evaluate the post-HRRP changes so as to accommodate potential changes at the hospital level in response to the announcement of HRRP penalties in 2010. We have specified hospital characteristics identified at baseline, and therefore interpret the estimated changes in readmission rates associated with HRRP as arising from direct and indirect (mediated) changes, including strategic hospital responses in making systematic changes in the profile of patients hospitalized.

Aside from studies comparing the readmissions experience of safety-net and non-safety net hospitals, several studies examining all hospitals together also found reductions in readmission rates following HRRP announcement in 2010. Studies by Zuckerman et al. [38], Figueroa et al. [39] and Chaiyachati et al. [40] found significantly large annual reductions in readmission rates. Several studies have indicated that upcoding of patient comorbidity status may account for a sizable proportion of the reduction in readmissions [41, 42]. As our study is based on Hospital Compared risk-adjusted readmission rates – that are based on diagnosis codes identified in both outpatient and inpatient claims data – our estimates are less susceptible to the over-estimation of reduction in readmissions.

The readmissions performance contrasts sharply with the penalty experience of safety-net hospitals relative to that of non-safety-net hospitals: during 2013-2016, safety-net hospitals were more likely to be penalized each year, more likely to be repeatedly penalized and have higher average penalty. Our findings on the penalty experience are consistent with those from prior studies, but present a more comprehensive overview of the penalty measures, spanning multiple years and national data. Using 2013 data alone, Joynt et al. [13] found that safety-net hospitals were more likely to be highly penalized compared to non-safety-net hospitals. Favini et al. [15] found larger mean penalty for safety-net hospitals in the initial years of HRRP (2013-2014) but similar penalty in the latter years (2015-2016). Using data from California, Gilman et al. [14] found safety-net hospitals to be more likely to be penalized compared to non-safety-net hospitals. In dollar terms, the Office of the Assistant Secretary for Planning and Evaluation also estimated the average penalty to be $191,000 for safety-net and $150,000 for non-safety-net hospitals (2011-2013) [43]. Although the difference in average amounts are relatively small, the adverse effect of the penalty may be greater among safety-net hospitals as they are likely to have a smaller “profit” (surplus) margin and rely more on Medicare revenues compared to non-profit hospitals [15].

This dichotomy in readmission rate performance and penalty experience validates and accentuates the concern of undue burden of HRRP penalties on safety-net hospitals. Cross-sectional differences in readmission rates across hospitals, which primarily underlie the penalty experience, may arise from systematic differences in patient profiles across hospitals. In the existing penalty determination algorithm, hospitals are penalized for unplanned readmissions that are unrelated to the index admission, despite the fact that such readmissions may not be associated with the care provided by the hospital [3]. Another concern is that the HRRP penalty formula does not adjust for factors that are outside the control of the hospital and increases the risk of readmissions. One study attributed roughly 60% of the variation in hospital readmission rates to community-level factors [44]. Joynt et al. estimated an enhanced risk adjustment model adding patient neighborhood deprivation indicator and found that readmissions performance of safety-net hospitals improved and that of other hospitals worsened [45]. Other studies have also highlighted the role of socioeconomic and environmental factors such as income, living status, social support, education, employment status, home stability, and risk behaviors in explaining a portion of the variation in readmission rates [6–10]. Another study found racial/ethnic differences in the likelihood of readmissions for targeted conditions [46]. It found that among Medicare enrollees, African-Americans having an index hospitalization for AMI, heart failure, or pneumonia had a higher likelihood of readmissions compared to whites. Overall, our results lend support to the concerns about the lack of fairness of the current HRRP penalty formula, particularly toward safety-net hospitals [3].

In response to the widespread concerns raised early after the announcement of HRRP, the twenty-first Century Cures Act of 2016 introduced modifications to HRRP beginning in 2019, wherein all hospitals are stratified by the proportion of low socioeconomic status patients served – as quantified by the proportion of Medicare patients who were also eligible for Medicaid benefits – into five groups (quintile “peer groups”), and readmissions performance will be assessed based on intra-group differences [20]. This modification was informed by early findings from studies in response to the Improving Medicare Post-Acute Care Transformation (IMPACT) Act, 2014 [47–49]. While the impact of the peer-group based HRRP is awaited, Joynt et al. [45] obtained penalty estimates under the traditional and the peer-group based HRRP penalty algorithms and found that hospitals serving in disadvantaged neighborhoods experienced lower penalties under the peer-group based program. Alternative modifications to HRRP performance evaluation have also been proposed; in particular, the National Quality Forum has advocated for the inclusion of social risk factors in the risk-adjustment models for pay-for-performance programs [50]. Recommending against this inclusion, the Office of the Assistant Secretary for Planning and Evaluation (ASPE) raises the concern that “if social risk adjustment were to undercut incentives to address the systemic problems that affect vulnerable patients, it could move us further away from an equitable health system “ [51, 52]. Instead ASPE favors a broader agenda of directly tackling social risk factors through additional supports in ongoing health-related programs and social service programs.

We acknowledge several limitations of our study. First, as our study data is observational, the ability to obtain firm causal estimates is limited. However, the difference-in-differences design is aimed at adjusting for unobserved secular trends to better isolate the changes in readmission rates associated with the introduction of the HRRP program. The “parallel trends” test indicated that, prior to the announcement of HRRP in 2010, the pattern of changes in readmission rates were similar between safety-net and non-safety net hospitals [31]. We concede that this test was limited to only 2 years (2009, 2010) since Hospital Compare reporting of hospital readmission rates began in 2009. HRRP has been expanded to include other admission cohorts (e.g., COPD). However, we limited the study to the three selected conditions as these original conditions have been the focus of much of the literature on HRRP impact. Second, given the lack of a universal definition of safety-net hospitals, our findings may be sensitive to the measure we used. Our measure based on the DSH index has been commonly used in prior studies. We also performed sensitivity analysis using an alternative definition of safety net hospitals, based on the Medicaid caseload of hospital admissions, and found largely similar results. Third, a limitation of using Hospital Compare data is that they do not include information on the racial/ethnic composition of the patients at the hospital level, thereby limiting our ability to examine subgroups of hospitals based on minority share of patients. As Hospital Compare risk-adjusted readmission rates are based on all index admissions during rolling 3-year periods, we were unable to estimate changes during individual years. As we only included hospitals that continuously were reimbursed through IPPS and for which Hospital Compare readmissions data were reported, the study cohort may not be representative of all hospitals.

Based on data from the first 4 years of HRRP penalties and readmission performance, our study findings add to the growing evidence that the HRRP performance assessment and penalty formula place an unduly higher risk of penalty on safety-net hospitals. While several modifications to the program have been suggested – with the imminent modification of performance assessment based on peer subgroups in 2019 – a clearer understanding of the nature of the modifications, their likely impact and the relative merits is needed. In the interim, CMS should consider measures to limit the burdensome revenue loss from HRRP penalties on safety-net hospitals, and introduce initiatives to collaboratively guide and support such hospitals in improving care for socioeconomically disadvantaged patients.

## Supplementary Information

**Additional file 1: Table 1.** Data collection and reporting dates for 30-day risk adjusted readmissions. **Table 2.** Validity of difference-in-differences model estimates of change in 30-day readmissions associated with HRRP: Parallel trends test. **Table 3.** Difference-in-differences model estimates of change in 30-day readmissions associated with HRRP: Main model estimates. **Table 4.** Sensitivity Analysis: Difference-in-difference model estimates of change in 30-day readmission rates using alternative definition of safety-net hospitals using Medicaid share inpatient days. **Table 5.** Sensitivity Analysis: Difference-in-difference model estimates of change in 30-day readmission rates using hospital fixed effects specification. **Figure 3.** Distribution of number of years of penalty under HRRP by safety-net status, 2013-2016.

## Data Availability

The datasets used and/or analysed during the current study are available from the corresponding author on reasonable request. We used publicly available nationwide hospital level data for 2009-2016 from the Centers for Medicare and Medicaid Services (CMS) Hospital Compare program http://www.medicare.gov/hospitalcompare/search.html;2018, CMS Final Impact Rule https://www.cms.gov/Medicare/Medicare-Fee-for-Service-Payment/AcuteInpatientPPS/Historical-Impact-Files-for-FY-1994-through-Present.html;2017 and the American Hospital Association Annual Survey (www.ahadata.com).

## Abbreviations

HRRP: Medicare’s Hospital Readmissions Reduction Program
CMS: Centers for Medicare and Medicaid Services
AMI: Acute Myocardial Infarction
HF: Heart Failure
IPPS: Inpatient Prospective Payment System
DSH: Disproportionate Share Hospital
