# Supplementary material for: Readmissions performance and penalty experience of safety-net hospitals under Medicare’s Hospital Readmissions Reduction Program
Source: BMC Health Serv Res. 2022 Mar 15;22:338. doi: 10.1186/s12913-022-07741-9 (PMC8922916; doi:10.1186/s12913-022-07741-9)
Supplement: Supplementary file 1 — Additional file 1: Table 1. Data collection and reporting dates for 30-day risk adjusted readmissions. Table 2. Validity of difference-in-differences model estimates of change in 30-day readmissions associated with HRRP: Parallel trends test. Table 3. Difference-in-differences model estimates of change in 30-day readmissions associated with HRRP: Main model estimates. Table 4. Sensitivity Analysis: Difference-in-difference model estimates of change in 30-day readmission rates using alternative definition of safety-net hospitals using Medicaid share inpatient days. Table 5. Sensitivity Analysis: Difference-in-difference model estimates of change in 30-day readmission rates using hospital fixed effects specification. Figure 3. Distribution of number of years of penalty under HRRP by safety-net status, 2013-2016. [file 12913_2022_7741_MOESM1_ESM.docx]

**Readmissions Performance and Penalty Experience of Safety-Net Hospitals under Medicare’s Hospital Readmissions Reduction Program**

**Additional file 1**

**Table 1: Data collection and reporting dates for 30-day risk adjusted readmissions**

| **Data collection period** | **Data reporting period** | **Analysis period** |
| --- | --- | --- |
| 7/1/2005-6/30/2008 | 2009 | Pre-period |
| 7/1/2006-6/30/2009 | 2010 |  |
| 7/1/2007-6/30/2010 | 2011 | Washout period |
| 7/1/2008-6/30/2011 | 2012 |  |
| 7/1/2009-6/30/2012 | 2013 |  |
| 7/1/2010-6/30/2013 | 2014 | Post-period |
| 7/1/2011-6/30/2014 | 2015 |  |
| 7/1/2012-6/30/2015 | 2016 |  |

Note:

1) The post-period for the pneumonia cohort ends in 2015, since the criteria for identifying pneumonia admission changed in 2016.

**Table 2: Validity of difference-in-differences model estimates of change in 30-day readmissions associated with HRRP: Parallel trends test**

|  | Acute myocardial infarction | Heart failure | Pneumonia |
| --- | --- | --- | --- |
| Safety-net hospitals | 0.55*** | 1.08*** | 0.63*** |
|  | (0.09) | (0.12) | (0.10) |
| Post 2010 | 0.05* | 0.26*** | 0.20*** |
|  | (0.03) | (0.03) | (0.02) |
| Safety-net hospitals x Post 2010 | -0.01 | -0.10 | -0.09* |
|  | (0.06) | (0.06) | (0.05) |
| Teaching hospital | 0.28** | 0.30* | 0.56*** |
|  | (0.12) | (0.16) | (0.12) |
| Government non-federal (ref: not-for-profit) | 0.19 | 0.52*** | 0.30*** |
|  | (0.12) | (0.14) | (0.11) |
| For-profit (ref: not-for-profit) | 0.12 | 0.63*** | 0.33*** |
|  | (0.09) | (0.13) | (0.10) |
| Bed size: 100-199 (ref: <100) | 0.08 | 0.24* | 0.16 |
|  | (0.13) | (0.13) | (0.10) |
| Bed size: >=200 (ref: <100) | 0.01 | -0.09 | 0.16* |
|  | (0.12) | (0.13) | (0.10) |
| Midwest (ref: Northeast) | -0.38*** | -0.76*** | -0.58*** |
|  | (0.11) | (0.14) | (0.12) |
| South (ref: Northeast) | -0.89*** | -1.22*** | -1.06*** |
|  | (0.10) | (0.13) | (0.11) |
| West (ref: Northeast) | -1.21*** | -1.65*** | -1.44*** |
|  | (0.11) | (0.15) | (0.12) |
| N | 2,890 | 3,748 | 3,790 |

Notes:

1) Safety-net hospitals: hospitals that fall in the top quartile of the Disproportionate Share Hospital (DSH) index; non-safety-net hospitals: hospitals in the bottom three quartiles of the DSH index.

2) Estimates from random effects model reported; heteroscedasticity-robust standard errors clustered at the hospital level; model includes year dummies.

3) * p<0.10; ** p<0.05; *** p<0.01

4) Standard errors in parenthesis.

**Table 3: Difference-in-differences model estimates of change in 30-day readmissions associated with HRRP: Main model estimates**

|  | Acute myocardial infarction | Heart failure | Pneumonia |
| --- | --- | --- | --- |
| Safety-net hospitals | 0.49*** | 1.01*** | 0.59*** |
|  | (0.09) | (0.11) | (0.09) |
| Post 2014 | -2.97*** | -2.42*** | -0.76*** |
|  | (0.04) | (0.06) | (0.04) |
| Safety-net hospitals x Post 2014 | -0.08 | -0.14 | -0.23*** |
|  | (0.09) | (0.10) | (0.09) |
| Teaching hospital | 0.24*** | 0.17 | 0.43*** |
|  | (0.08) | (0.11) | (0.09) |
| Government non-federal (ref: not-for-profit) | 0.13 | 0.30*** | 0.10 |
|  | (0.08) | (0.10) | (0.08) |
| For-profit (ref: not-for-profit) | 0.24*** | 0.54*** | 0.25*** |
|  | (0.06) | (0.09) | (0.07) |
| Bed size: 100-199 (ref: <100) | 0.18** | 0.17* | 0.14** |
|  | (0.09) | (0.09) | (0.07) |
| Bed size: >=200 (ref: <100) | 0.15* | -0.02 | 0.16** |
|  | (0.09) | (0.09) | (0.07) |
| Midwest (ref: Northeast) | -0.29*** | -0.79*** | -0.39*** |
|  | (0.07) | (0.10) | (0.08) |
| South (ref: Northeast) | -0.51*** | -0.90*** | -0.69*** |
|  | (0.07) | (0.10) | (0.08) |
| West (ref: Northeast) | -0.80*** | -1.34*** | -1.04*** |
|  | (0.08) | (0.11) | (0.09) |
| N | 7225 | 9370 | 7580 |

Notes:

1) Safety-net hospitals: hospitals that fall in the top quartile of the Disproportionate Share Hospital (DSH) index; non-safety-net hospitals: hospitals in the bottom three quartiles of the DSH index.

2) Estimates from random effects model reported; heteroscedasticity-robust standard errors clustered at the hospital level; model includes year dummies.

3) * p<0.10; ** p<0.05; *** p<0.01

4) Standard errors in parenthesis.

**Table 4: Sensitivity Analysis: Difference-in-difference model estimates of change in 30-day readmission rates using alternative definition of safety-net hospitals using Medicaid share inpatient days**

|  | Acute myocardial infarction | Heart failure | Pneumonia |
| --- | --- | --- | --- |
| Safety-net hospitals | 0.23*** | 0.59*** | 0.26*** |
|  | (0.08) | (0.11) | (0.09) |
| Post 2014 | -2.97*** | -2.41*** | -1.19*** |
|  | (0.04) | (0.06) | (0.04) |
| Safety-net hospitals x Post 2014 | -0.08 | -0.15 | -0.20** |
|  | (0.09) | (0.10) | (0.08) |
| Teaching hospital | 0.32*** | 0.33*** | 0.53*** |
|  | (0.08) | (0.11) | (0.09) |
| Government non-federal (ref: not-for-profit) | 0.15* | 0.32*** | 0.14* |
|  | (0.08) | (0.11) | (0.08) |
| For-profit (ref: not-for-profit) | 0.28*** | 0.66*** | 0.30*** |
|  | (0.06) | (0.09) | (0.07) |
| Bed size: 100-199 (ref: <100) | 0.20** | 0.17* | 0.15** |
|  | (0.09) | (0.09) | (0.07) |
| Bed size: >=200 (ref: <100) | 0.21** | 0.02 | 0.19*** |
|  | (0.09) | (0.09) | (0.07) |
| Midwest (ref: Northeast) | -0.32*** | -0.82*** | -0.40*** |
|  | (0.07) | (0.11) | (0.09) |
| South (ref: Northeast) | -0.49*** | -0.84*** | -0.66*** |
|  | (0.07) | (0.10) | (0.08) |
| West (ref: Northeast) | -0.75*** | -1.21*** | -0.96*** |
|  | (0.08) | (0.11) | (0.09) |
| N | 7225 | 9370 | 7580 |

Notes:

1) Safety-net hospitals: hospitals that fall in the top quartile of Medicaid share inpatient days; non-safety-net hospitals: hospitals in the bottom three quartiles of the Medicaid share inpatient days.

2) Models estimated are similar to that in Table 3. Estimates from random effects model reported; heteroscedasticity-robust standard errors clustered at the hospital level; model includes year dummies.

3) * p<0.10; ** p<0.05; *** p<0.01

4) Standard errors in parenthesis.

**Table 5: Sensitivity Analysis: Difference-in-difference model estimates of change in 30-day readmission rates using hospital fixed effects specification**

|  | Acute myocardial infarction | Heart failure | Pneumonia |
| --- | --- | --- | --- |
| Post 2014 | -2.99*** | -2.43*** | -1.18*** |
|  | (0.04) | (0.05) | (0.04) |
| Safety-net hospitals x Post 2014 | 0.003 | -0.08 | -0.21** |
|  | (0.07) | (0.08) | (0.07) |
| N | 7225 | 9370 | 7580 |

Notes:

1) Models similar to that in Table 3 are estimated now with hospital-level fixed effects specification.

2) Note that hospital-level covariates (e.g., safety-net hospital status, teaching hospital, region) are excluded as they are time-invariant.

3) * p<0.10; ** p<0.05; *** p<0.01

4) Standard errors in parenthesis.

**Figure 3: Distribution of number of years of penalty under HRRP by safety-net status, 2013-2016**

Notes:

1) Safety-net hospitals: hospitals that fall in the top quartile of the Disproportionate Share Hospital (DSH) index; non-safety-net hospitals: hospitals in the bottom three quartiles of the DSH index.

2) Mean number of years of penalty under HRRP for safety-net hospitals: 3.50 (CI: 3.45, 3.54); non-safety-net hospitals: 3.17 (CI: 3.14, 3.20); difference: 0.33 (p-value <0.001).
